# Validation of Risk Scores for Predicting Atrial Fibrillation Detected After Stroke Based on an Electronic Medical Record Algorithm: A Registry-Claims-Electronic Medical Record Linked Data Study

**DOI:** 10.3389/fcvm.2022.888240

**Published:** 2022-04-29

**Authors:** Cheng-Yang Hsieh, Hsuan-Min Kao, Kuan-Lin Sung, Luciano A. Sposato, Sheng-Feng Sung, Swu-Jane Lin

**Affiliations:** ^1^Department of Neurology, Tainan Sin Lau Hospital, Tainan City, Taiwan; ^2^School of Pharmacy, Institute of Clinical Pharmacy and Pharmaceutical Sciences, College of Medicine, National Cheng Kung University, Tainan City, Taiwan; ^3^Division of Geriatrics, Department of Internal Medicine, Ditmanson Medical Foundation Chia-Yi Christian Hospital, Chiayi City, Taiwan; ^4^School of Medicine, National Taiwan University, Taipei City, Taiwan; ^5^Department of Clinical Neurological Sciences, Schulich School of Medicine and Dentistry, Western University, London, ON, Canada; ^6^Heart & Brain Laboratory, Western University, London, ON, Canada; ^7^Department of Epidemiology and Biostatistics and Anatomy and Cell Biology, Schulich School of Medicine and Dentistry, Western University, London, ON, Canada; ^8^Robarts Research Institute, Western University, London, ON, Canada; ^9^Lawson Health Research Institute, London, ON, Canada; ^10^Division of Neurology, Department of Internal Medicine, Ditmanson Medical Foundation Chia-Yi Christian Hospital, Chiayi City, Taiwan; ^11^Department of Nursing, Min-Hwei Junior College of Health Care Management, Tainan City, Taiwan; ^12^Department of Pharmacy Systems, Outcomes and Policy, College of Pharmacy, University of Illinois at Chicago, Chicago, IL, United States

**Keywords:** atrial fibrillation, external validation, ischemic stroke, prediction, risk score

## Abstract

**Background:**

Poststroke atrial fibrillation (AF) screening aids decisions regarding the optimal secondary prevention strategies in patients with acute ischemic stroke (AIS). We used an electronic medical record (EMR) algorithm to identify AF in a cohort of AIS patients, which were used to validate eight risk scores for predicting AF detected after stroke (AFDAS).

**Methods:**

We used linked data between a hospital stroke registry and a deidentified database including EMRs and administrative claims data. EMR algorithms were constructed to identify AF using diagnostic and medication codes as well as free clinical text. Based on the optimal EMR algorithm, the incidence rate of AFDAS was estimated. The predictive performance of 8 risk scores including AS5F, C_2_HEST, CHADS_2_, CHA_2_DS_2_-VASc, CHASE-LESS, HATCH, HAVOC, and Re-CHARGE-AF scores, were compared using the C-index, net reclassification improvement, integrated discrimination improvement, calibration curve, and decision curve analysis.

**Results:**

The algorithm that defines AF as any positive mention of AF-related keywords in electrocardiography or echocardiography reports, or presence of diagnostic codes of AF was used to identify AF. Among the 5,412 AIS patients without known AF at stroke admission, the incidence rate of AFDAS was 84.5 per 1,000 person-year. The CHASE-LESS and AS5F scores were well calibrated and showed comparable C-indices (0.741 versus 0.730, *p* = 0.223), which were significantly higher than the other risk scores.

**Conclusion:**

The CHASE-LESS and AS5F scores demonstrated adequate discrimination and calibration for predicting AFDAS. Both simple risk scores may help select patients for intensive AF monitoring.

## Introduction

Stroke is one of the leading causes of death and long-term disability in adults globally (1). In the past three decades, the absolute numbers of incident and prevalent strokes have increased by 70 and 85 percent, respectively (1). Moreover, the rate of stroke recurrence has remained unchanged over the last decade, particularly for cardioembolic strokes (2). Hence, current stroke guidelines highlight the importance of identifying potential sources of embolism to determine the optimal strategy for secondary stroke prevention (3).

Atrial fibrillation (AF) is the most common arrhythmia in clinical practice and a major source of cardioembolism. Oral anticoagulation can effectively reduce the risk of stroke recurrence in patients with known AF (KAF) (3). In stroke patients without KAF, applying sequential monitoring strategies including the use of implantable loop recorders could lead to an additional yield of 24% detection of AF after stroke (4), thereby allowing prompt secondary prevention. In the context of limited medical resources, it is reasonable to reserve aggressive poststroke AF search and electrocardiographic monitoring for selected patients at a high risk of AF detection based on their clinical characteristics (5).

To date, more than a dozen of risk scores have been developed or validated to assess the risk of AF detection after acute ischemic stroke (AIS) or transient ischemic attack (TIA) (6, 7). These risk scores vary considerably in their component variables, outcome definition, and ease of implementation (Supplementary Table 1). Besides patients’ demographics and comorbidities, many of these risk scores require additional diagnostic work-up to obtain the necessary components, such as blood, electrocardiography (ECG), and echocardiography markers (6). Due to the limitation of medical resources, not all risk scores could be applied equally in all healthcare settings by all practitioners. Moreover, most of currently available risk scores have not been externally validated or undergone head-to-head comparison (6), thus limiting their generalizability and clinical utility.

Therefore, this study aimed to assess the performance of 8 risk scores for predicting atrial fibrillation detected after stroke (AFDAS) based on routinely collected clinical variables.

## Materials and Methods

### Data Sources

This retrospective study was conducted in a 1,000-bed teaching hospital with approximately 650 stroke admissions annually. In this study, we used both data from the hospital stroke registry and the Ditmanson Research Database (DRD). Patients in both datasets are linked by unique identifiers. The hospital stroke center set up its stroke registry in 2007, conforming to the design of the nationwide Taiwan Stroke Registry (8). The stroke registry prospectively registers all cases of stroke admitted within 10 days of symptom onset. Data regarding the demographics, stroke etiology, risk factor profiles, stroke severity based on the National Institutes of Health Stroke Scale (NIHSS), acute treatment, and outcomes are routinely collected by trained stroke case managers. The DRD is a deidentified research-based database integrating electronic medical records (EMRs) and administrative claims data. It currently holds clinical information of over 1.4 million patients cared at the Ditmanson Medical Foundation Chia-Yi Christian Hospital from 2006 to 2021, including approximately 0.6 million inpatient records and 21.5 million outpatient records. We linked the stroke registry to the DRD using a unique encrypted patient identifier. Information regarding past medical history before admission and clinical variables collected during the index stroke hospitalization were obtained from the stroke registry. Billing information and medical records from 2 years before to 1 year after the index hospitalization were extracted from the DRD (Supplementary Figure 1).

The study protocol was approved by the Ditmanson Medical Foundation Chia-Yi Christian Hospital Institutional Review Board (IRB2020135). The requirement for informed consent was waived because of the retrospective design. The study protocol conforms to the ethical guidelines of the 1975 Declaration of Helsinki.

### Study Population

The study population selection is shown in Supplementary Figure 2. The first hospitalization for AIS for each patient between Oct 2007 and Sep 2020 was identified from the stroke registry. Patients with an in-hospital stroke or whose data in the DRD could not be linked were excluded. The primary study outcome was AFDAS. In accordance with the updated definition of AFDAS, patients who had KAF before the index stroke hospitalization or whose admission ECG showed AF were further excluded (9). All patients were followed until the occurrence of AFDAS or death, the last visit within 1 year after the index stroke, or February 28, 2021, whichever occurred first.

### Primary Outcome Ascertainment

Atrial fibrillation was identified in the EMR by adapting a previously validated AF-specific EMR algorithm based mainly on diagnostic and billing codes (10). The major modification was the use of natural language processing to detect the presence of keywords in physicians’ notes as well as ECG and echocardiography reports. Free text in the notes and reports were screened for mentions of AF-related keywords including “atrial fibrillation,” “AF,” “Afib,” and “PAF.” NegEx was used to determine whether the mention was positive or negative (11). The modified EMR algorithms comprise different components, which are further combined into composite algorithms as shown in Supplementary Table 2. By applying these algorithms, the AF status of each patient before discharge from the stroke hospitalization was determined using all available information up until the discharge date. We tested the discriminatory ability of each component and composite algorithm by using the manual screening of EMRs of 1,000 randomly selected patients (Supplementary Figure 2) as the gold standard. EMRs were reviewed by two experienced stroke neurologists. The degree of agreement of AF status between clinicians was assessed using the Kappa statistic. The performance of each algorithm was evaluated by estimating the sensitivity, specificity, positive predictive value (PPV), and negative predictive value (NPV). We finally chose the best performing algorithm based on their area under the receiver operating characteristic curve (AUC) and we used it for identifying AFDAS in the whole cohort.

### Risk Scores

We conducted a comprehensive search of the literature to identify articles reporting the derivation or validation of risk scores for predicting AFDAS published until December 2021. A total of 22 risk scores were identified (Supplementary Table 1). These risk scores vary in the types of component variables, which can be categorized into demographics, comorbidities, clinical markers, laboratory markers, ECG markers, echocardiography markers, and imaging markers. Considering the trade-offs between predictive performance and ease of implementation, we assessed 8 of the risk scores. They are based on readily available variables and are deemed suitable for routine clinical use (Supplementary Table 3).

The AS5F score was developed and validated using data from three prospective studies performing prolonged Holter-ECG monitoring in patients hospitalized for AIS or TIA (12). It is a very simple clinical score consisting of only age and NIHSS score. The C_2_HEST score was originally proposed to predict incident AF among Asian general populations (13). It demonstrated moderately good predictive abilities in predicting the risk of incident AF as well as death and hospitalization in patients with heart failure with preserved ejection fraction (14). It also performed well in discriminating the risk of developing incident AF in a French population hospitalized for AIS (15). The well-known CHADS_2_ and CHA_2_DS_2_-VASc scores were formerly developed for predicting stroke in patients with AF (16, 17) and were later found to predict incident AF in a large cohort of patients hospitalized for AIS collected from the French national administrative database (18). The CHASE-LESS score was developed by using data from patients hospitalized for AIS from the Taiwan National Health Insurance claims database and included both positive and negative items in the score calculation (19). The HATCH score was initially developed to identify patients who are likely to progress from paroxysmal to persistent forms of AF within 1 year (20) and was later found to predict new-onset AF in the general population (21) and patients with AIS (22). The HAVOC score was established by using data from a hospital-based research database where predictor and outcome variables were identified from diagnostic codes (23). It only included patients with cryptogenic stroke or TIA in the study cohort. The final risk model is a modification of the original CHARGE-AF model, which was intended to predict incident AF in the general population (24). The Re-CHARGE-AF model exhibited an excellent discrimination and good calibration in a cohort of AIS patients (7).

### Statistical Analysis

Categorical variables were described with counts and percentages. Continuous variables were reported as means with standard deviations for normally distributed data and the median and interquartile range for non-normally distributed data. Differences between groups were compared by Chi-square tests for categorical variables and *t*-tests or Mann–Whitney U tests for continuous variables, as appropriate.

The incidence rate of AFDAS was expressed as events per 1,000 person-years. To assess the prediction performance of each risk score, Cox proportional hazard regression analyses were performed by entering each risk score as a continuous variable. After fitting the model, the proportional hazards assumption was assessed using the Schoenfeld test. Harrell’s concordance index (C-index) was calculated for evaluation and comparison of model performance. The optimism-adjusted C-index was also estimated using bootstrapping with 250 replications of patients sampled with replacement. The value of C-index ranges from 0.5 to 1.0, with 0.5 indicating random chance and 1 indicating perfect concordance. In clinical application, a C-index value of 0.7 or higher is considered acceptable discrimination (25). In addition to the C-index, the improvement in predictive performance was assessed by calculating the continuous net reclassification improvement (NRI) and integrated discrimination improvement (IDI) indices (26, 27). Unlike categorical NRI, continuous NRI does not require prespecified risk categories. It measures upward and downward changes in an event’s predicted probabilities. The IDI index represents the difference in discrimination slopes, which quantity the difference between the average of the predicted probabilities of an event for those with events and the corresponding average for those without events (26, 27). Higher NRI and IDI values indicate better discrimination.

Calibration was assessed by plotting the predicted versus actual risk of AFDAS for quintiles of the predicted probability. Bootstraps with 1,000 times of resamples with replacement were applied to the calibration curve. In a well calibrated model, the data points in the calibration curve should be close to the 45-degree diagonal line. Finally, to assess the clinical usefulness of the risk scores, decision curve analysis was used to estimate the net benefit, i.e., the ability to make better decisions with a model than without it (28).

All statistical analyses were performed using Stata 15.1 (StataCorp, College Station, TX, United States) and R version 4.1.1 (R Foundation for Statistical Computing, Vienna, Austria). Pairwise comparisons of C-indices were performed with the STATA procedures “somersd” and “lincom” (29). The optimism-adjusted C-index was estimated using the R package “rms”. The NRI and IDI indices were calculated using the R package “survIDINRI”. The calibration plot was performed using the R package “riskRegression.” Two-tailed *p*-values were considered statistically significant at <0.05.

## Results

### Optimal Atrial Fibrillation-Specific Electronic Medical Record Algorithm

Of the 1,000 patients whose EMRs were manually reviewed, 169 patients were adjudicated as having AF. The Kappa statistic between the reviewers was 0.91 indicating excellent interrater agreement. Supplementary Table 4 lists the performance of various AF-specific EMR algorithms. The highest AUC (0.990) was attained by the composite algorithm that defines AF as any positive mention of AF-related keywords in ECG or echocardiography reports, or presence of diagnostic codes of AF (AF-D in Supplementary Tables 2, 4). Its sensitivity, specificity, PPV, and NPV were 98.2, 99.8, 98.8, and 99.6%, respectively. Therefore, this algorithm was chosen for identifying KAF and AFDAS in the whole cohort.

### Characteristics of the Study Population

We identified 6,523 patients hospitalized for AIS from the stroke registry (Supplementary Figure 2). After excluding 199 patients with in-hospital stroke and 2 patients with unavailable DRD data, the remaining 6,322 patients were successfully linked to the DRD. Among them, 910 patients were determined to have KAF at stroke admission and were thus excluded from the study population. During the follow-up of the remaining 5,412 patients, 316 (5.8%) patients were identified as having AFDAS after a median follow-up of 10.6 months. Each patient had a mean of 3.1 hospital visits per month during the follow-up period. The incidence rate of AFDAS was 84.5 per 1,000 person-year. Table 1 shows the characteristics of the study population. Patients with AFDAS were older, more likely to be female, and tended to have coronary artery disease, prior stroke or TIA, peripheral artery disease, valve disease, and chronic obstructive pulmonary disease, as well as lower diastolic blood pressure, height, weight, and body mass index than those with no AFDAS. They also had significantly higher NIHSS score and AFDAS risk scores but were less likely to have diabetes and hyperlipidemia.

**TABLE 1:** Baseline characteristics of the study population.

Characteristic	All (*N* = 5412)	AFDAS (*N* = 316)	No AFDAS (*N* = 5096)	*P*
Age, mean (SD)	68.0 (3.8)	76.5 (10.1)	67.4 (12.6)	<0.001
Female	2085 (38.5)	159 (50.3)	1926 (37.8)	<0.001
Hypertension	4261 (78.7)	257 (81.3)	4004 (78.6)	0.245
Diabetes mellitus	2406 (44.5)	123 (38.9)	2283 (44.8)	0.041
Hyperlipidemia	3217 (59.4)	137 (43.4)	3080 (60.4)	<0.001
Coronary artery disease	522 (9.6)	52 (16.5)	470 (9.2)	<0.001
Congestive heart failure	136 (2.5)	13 (4.1)	123 (2.4)	0.061
Prior stroke or TIA	1203 (22.2)	89 (28.2)	1114 (21.9)	0.009
Peripheral artery disease	144 (2.7)	20 (6.3)	124 (2.4)	<0.001
Valve disease	24 (0.4)	6 (1.9)	18 (0.4)	<0.001
COPD	425 (7.9)	39 (12.3)	386 (7.6)	0.002
Hyperthyroidism	32 (0.6)	4 (1.3)	28 (0.5)	0.107
Smoking (current)	2295 (42.4)	85 (26.9)	2210 (43.4)	<0.001
SBP, median (IQR)	161 (144–185)	158 (143–183)	162 (144–186)	0.107
DBP, median (IQR)	91 (80–103)	88 (76–101)	91 (80–103)	0.001
BMI, median (IQR)	24.7 (22.1–27.3)	23.5 (20.9–25.9)	24.7 (22.3–27.3)	<0.001
Height, median (IQR)	160 (153–165)	158 (152–165)	160 (153–165)	0.028
Weight, median (IQR)	63 (55–71)	58 (50–67)	63 (55–71)	<0.001
NIHSS, median (IQR)	5 (2–8)	8 (4–19)	5 (2–8)	<0.001
AS5F, median (IQR)	66 (58–74)	76 (68–83)	65 (57–73)	<0.001
C_2_HEST, median (IQR)	1 (1–3)	3 (1–3)	1 (1–3)	<0.001
CHADS_2_, median (IQR)	2 (1–3)	2 (2–3)	2 (1–3)	<0.001
CHA_2_DS_2_-VASc, median (IQR)	4 (3–5)	5 (4–6)	4 (3–5)	<0.001
CHASE-LESS, median (IQR)	6 (4–7)	8 (6–10)	6 (4–7)	<0.001
HATCH, median (IQR)	1 (1–2)	2 (1–3)	1 (1–2)	<0.001
HAVOC, median (IQR)	2 (2–4)	4 (2–4)	2 (2–4)	<0.001
Re-CHARGE-AF, median (IQR)	2.33 (1.66–2.90)	2.87 (2.32–3.38)	2.30 (1.63–2.86)	<0.001

*Data are numbers (percentage) unless specified otherwise.*

*AFDAS, atrial fibrillation detected after stroke; BMI, body mass index; COPD, chronic obstructive pulmonary disease; DBP, diastolic blood pressure; IQR, interquartile range; NIHSS, National Institutes of Health Stroke Scale; SBP, systolic blood pressure; SD, standard deviation; TIA, transient ischemic attack.*

### Performance of Risk Scores

Table 2 shows the performance of the eight risk scores. All the risk scores were significantly associated with the risk of AFDAS. However, their C-indices varied considerably, ranging from 0.584 to 0.741. Only the CHASE-LESS and AS5F scores achieved a C-index value greater than 0.7. Supplementary Table 5 shows the *p*-values of pairwise comparisons of C-indices between the risk scores. The C-indices of the CHASE-LESS and AS5F scores were comparable (0.741 versus 0.730, *p* = 0.223) but were significantly higher than those of the C_2_HEST, CHADS_2_, CHA_2_DS_2_-VASc, HATCH, HAVOC, and Re-CHARGE-AF scores (all *p* < 0.001 except for AS5F versus Re-CHARGE-AF *p* = 0.002). Both NRI and IDI analyses (Table 3) demonstrated that the CHASE-LESS and AS5F scores more accurately predicted AFDAS than did the other 6 risk scores. The CHASE-LESS score significantly improved reclassification over the C_2_HEST, CHADS_2_, CHA_2_DS_2_-VASc, HATCH, HAVOC, and Re-CHARGE-AF scores with NRI values of 27.1, 26.6, 24.5, 32.7, 30.5, and 20.2%, respectively. The corresponding NRI values for the AS5F score were 29.9, 37.4, 28.7, 34.9, 35.3, and 18.8%, respectively. The IDI indices also showed significant improvement in discrimination slopes for the CHASE-LESS and AS5F scores compared to the other 6 risk scores.

**TABLE 2:** Performance of risk scores for prediction of atrial fibrillation detected after stroke.

Risk score	HR (95% CI)	*P*	Schoenfeld’s global test	C-index (95% CI)	Bootstrapped C-index (95% CI)
AS5F	1.08 (1.07–1.09)	<0.001	0.050	0.730 (0.705–0.756)	0.731 (0.701–0.752)
C_2_HEST	1.54 (1.43–1.67)	<0.001	0.002	0.656 (0.625–0.687)	0.657 (0.624–0.684)
CHADS_2_	1.28 (1.18–1.39)	<0.001	0.010	0.584 (0.553–0.615)	0.586 (0.554–0.616)
CHA_2_DS_2_-VASc	1.35 (1.27–1.44)	<0.001	0.007	0.641 (0.611–0.671)	0.642 (0.611–0.668)
CHASE-LESS	1.44 (1.38–1.51)	<0.001	0.736	0.741 (0.715–0.768)	0.742 (0.713–0.765)
HATCH	1.37 (1.26–1.48)	<0.001	0.016	0.609 (0.578–0.640)	0.611 (0.579–0.639)
HAVOC	1.29 (1.22–1.36)	<0.001	0.003	0.636 (0.606–0.667)	0.638 (0.605–0.664)
Re-CHARGE-AF	2.32 (2.02–2.67)	<0.001	0.671	0.691 (0.662–0.718)	0.691 (0.662–0.718)

*CI, confidence interval; HR, hazard ratio.*

**TABLE 3:** Net reclassification improvement (NRI) and integrated discrimination improvement (IDI) indices between each pair of two different risk scores.

Risk score	C_2_HEST	CHADS_2_	CHA_2_DS_2_-VASc	CHASE-LESS	HATCH	HAVOC	Re-CHARGE-AF
AS5F	+29.9%/+3.6% (<0.001/<0.001)	+37.4%/+5.8% (<0.001/<0.001)	+28.7%/+4.6% (<0.001/<0.001)	−4.3%/−2.0% (0.598/0.047)	+34.9%/+5.0% (<0.001/<0.001)	+35.3%/+4.7% (<0.001/<0.001)	+18.8%/+2.7% (0.020/0.013)
C_2_HEST	–	+23.7%/+2.2% (<0.001/<0.001)	−2.8%/ + 1.0% (0.791/0.109)	−27.1%/−5.6% (<0.001/<0.001)	+22.4%/+1.4% (0.010/0.020)	+27.9%/ + 1.1% (<0.001/<0.001)	−14.2%/−0.9% (0.027/0.173)
CHADS_2_	–	–	−21.3%/−1.2% (<0.001/<0.001)	−26.6%/−7.8% (<0.001/<0.001)	−14.8%/−0.8% (0.020/0.007)	−7.7%/−1.1% (0.186/0.040)	−22.0%/−3.2% (<0.001/<0.001)
CHA_2_DS_2_-VASc	–	–	–	−24.5%/−6.6% (<0.001/<0.001)	+11.8%/+0.4% (0.020/0.306)	+1.2%/+0.1% (0.711/0.844)	−18.5%/−2.0% (0.027/0.027)
CHASE-LESS	–	–	–	–	+32.7%/+7.0% (<0.001/<0.001)	+30.5%/+6.7% (<0.001/<0.001)	+20.2%/+4.6% (0.020/<0.001)
HATCH	–	–	–	–	–	+4.1%/−0.3% (0.784/0.664)	−20.9%/−2.3% (0.007/0.007)
HAVOC	–	–	–	–	–	–	−18.2%/−2.1% (<0.001/<0.001)

*Each cell contains NRI/IDI (p-value/p-value) of the risk score listed in the first column versus the risk score listed in the first row.*

*IDI, integrated discrimination improvement; NRI, net reclassification improvement.*

**FIGURE 1:**
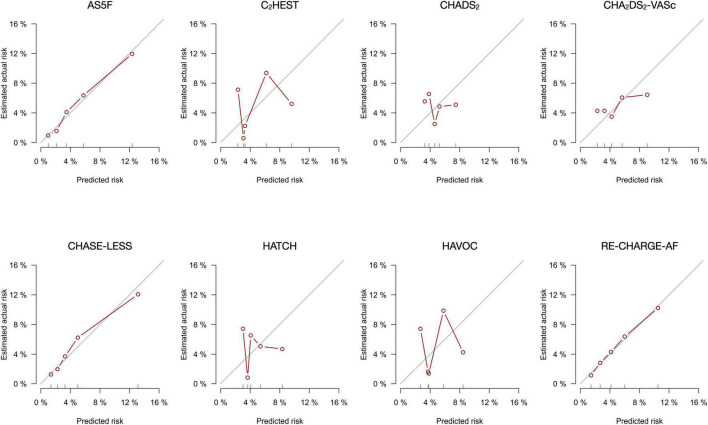
Calibration plots for the eight risk scores for predicting the risk of atrial fibrillation detected after stroke within 1 year of stroke.

**FIGURE 2:**
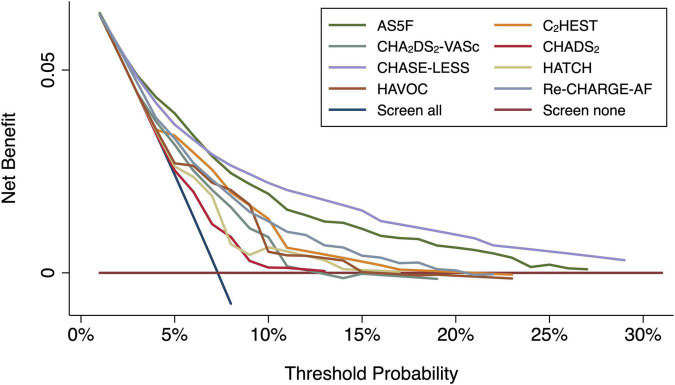
Decision curve analysis graph for the risk scores. The *y*-axis represents the net benefit, and the *x*-axis represents the threshold probability. The diagonal blue line (screen all) indicates the net benefit assuming all patients are at risk for atrial fibrillation detected after stroke, while the horizontal brown line (screen none) assumes none are at risk for atrial fibrillation detected after stroke.

The calibration plots (Figure 1) for the AS5F, CHASE-LESS, and Re-CHARGE-AF scores displayed a close agreement between the predicted and estimated actual risks, indicating good calibration. By contrast, the calibration curve of the C_2_HEST, CHADS_2_, CHA_2_DS_2_-VASc, HATCH, and HAVOC scores demonstrated a substantial deviation from the 45-degree diagonal line. The decision curve analysis graph (Figure 2) shows that the CHASE-LESS and AS5F scores provide a larger net benefit across ranges of threshold probabilities than the other risk scores. The CHASE-LESS score adds more benefit than the AS5F score if the threshold probability is between approximately 7.5 and 30% whereas the AS5F score has a higher net benefit if the threshold probability is between approximately 4 and 7.5%.

## Discussion

We assessed the performance of eight risk scores for predicting the risk of AFDAS and found that the CHASE-LESS and AS5F scores performed equally well and significantly better than the C_2_HEST, CHADS_2_, CHA_2_DS_2_-VASc, HATCH, HAVOC, and Re-CHARGE-AF scores.

### Performance of Risk Scores for Atrial Fibrillation Detected After Stroke

Supplementary Table 1 summarized the existing risk scores for predicting AFDAS. Although some risk scores achieved excellent performance with a C-index of 0.8 or higher in the derivation sample (30–35), most of them were not tested outside the derivation sample. Moreover, these scores need additional information, such as blood, echocardiography, and imaging markers, that may require extra effort to procure. Consequently, these risk scores are not very practical for routine clinical use.

On the other hand, despite being relatively easy to implement, six of the assessed risk scores in this study including the C_2_HEST, CHADS_2_, CHA_2_DS_2_-VASc, HATCH, HAVOC, and Re-CHARGE-AF scores had only modest predictive performance. Their C-indices were below the accepted 0.7 threshold necessary to have clinical relevance at the individual patient level. Previous studies have shown that the C-indices of the CHADS_2_ score ranged from 0.536 to 0.700 (12, 18, 19, 22, 36, 37) and those of the CHA_2_DS_2_-VASc score varied between 0.578 and 0.706 (15, 18, 19, 22, 36, 37), whereas the HATCH score attained C-indices of 0.612 and 0.653 (22). Only the C_2_HEST score achieved a C-index of 0.734 in the external validation cohort (15). This might be because all the six scores do not consider stroke severity, which was found to predict the diagnosis of AFDAS and improve the performance of risk scores (6, 22). By contrast, the CHASE-LESS and AS5F scores include the NIHSS score in the risk score formula and indeed performed satisfactorily.

Furthermore, most of the risk scores were not derived from a stroke population and some of them were originally developed for a different purpose. Consequently, the relationships between the predictors and outcome may be dissimilar across contexts of risk score application. For example, diabetes mellitus, one of the components of the CHADS_2_ and CHA_2_DS_2_-VASc scores, is in fact a negative rather than positive predictor of AFDAS (7, 19). Such a negative association was also observed in the case of incident AF after embolic stroke of undetermined source (38). As a result, both scores were found not very useful in predicting AFDAS (22, 36, 39). By contrast, diabetes mellitus has a negative coefficient in the CHASE-LESS and Re-CHARGE-AF scores, which might contribute to their better calibration.

### Clinical Significance of Atrial Fibrillation Detected After Stroke

Atrial fibrillation detected after stroke is a novel and distinct clinical entity. Compared to those with KAF, stroke patients with AFDAS have fewer vascular risk factors and comorbidities, fewer structural abnormalities of the heart such as left atrial enlargement, and are more likely to have strokes affecting the insular cortex (9). In addition to previously undiagnosed cardiogenic AF leading to stroke, stroke itself can cause transient neurogenic AF through inflammation, autonomic dysfunction, and stroke-induced heart injury (9, 40). Some AFDAS may even be just a bystander, rather than the cause of the stroke (41). However, we were unable to differentiate the phenotypes of AFDAS in the current study.

Although the risk of stroke recurrence of AFDAS is 26% lower than KAF (42), its risk is twice as high as the risk of stroke recurrence of no-AF (sinus rhythm or other non-AF rhythms) (9). The different risk of stroke recurrence between KAF and AFDAS may be because AFDAS is a heterogeneous mixture of high-burden (mainly cardiogenic) and low-burden (mainly neurogenic) AF phenotypes (40). In addition to stroke recurrence, AFDAS plays a significant role in many deleterious clinical outcomes. For example, AFDAS confirmed either during the stroke admission or during the outpatient follow-up period after discharge increases the risk of dementia by 78 and 74%, respectively, compared to no-AF (43). Furthermore, AFDAS raises the risk of all-cause death by 60% than no-AF (44).

### Application of Risk Scores

Poststroke AF screening is one of the research priorities identified by global experts regarding the detection of undiagnosed AF (45). The detection of AF poststroke generally dictates the necessity of long-term anticoagulation (46). The diagnosis of AF in the current study, as well as in many of the prior studies (Supplementary Table 1), was made in usual-care settings rather than clinical trials. In other words, AF was mostly documented using 12-lead ECG or 24-h Holter ECG. As such, the findings of this study are valid for relatively high-burden AFDAS detected on short-term monitoring rather than long-term strategies (47). Furthermore, this phenotype of AFDAS is likely to be more clinically relevant and benefits more from oral anticoagulation than low-burden AFDAS (41). Even though intensive or prolonged monitoring may greatly increase the yield of identification of subclinical AF (48), whether prolonged cardiac monitoring can decrease the risk of stroke recurrence is yet to be determined (49).

In view of this and to be resource efficient, intensive or prolonged ECG monitoring should be reserved for a selected population at a high risk of AFDAS. The European Society of Cardiology guidelines (50) also recommend that not all stroke patients are expected to benefit from prolonged ECG monitoring. Instead, they should be reserved for selected stroke patients without previous KAF such as those bearing a high risk of developing AF. Consequently, development and validation of AFDAS prediction tools are of high clinical significance and relevance. Some of the risk score development studies also established cutoff points to stratify patients into different risk groups (12, 19, 23), even though risk stratification is not meant as absolute criteria for selecting candidates for advanced cardiac monitoring (12). This study externally validated eight existing risk scores that are based on simple clinical parameters. The study findings can provide guidance to physicians in making clinical decisions in the context of medical resource allocation and patient preference, particularly in healthcare systems where the access to advanced cardiac monitoring is limited.

### Study Strengths and Limitations

This study performed a head-to-head comparison of eight AFDAS predicting scores widely used in clinical practice by using a large cohort of stroke patients with robustly validated outcomes. Some limitations are worth noting. First, patients in this study were traced through the hospital database. Because patients might receive a diagnosis of AF outside the study hospital, some degree of outcome misclassification is possible. Nevertheless, the relatively frequent visits to the study hospital (>3 visits per month) by the patients may have mitigated this problem. Second, this is a single-site study and therefore the study findings may not be generalizable to other settings. Further validation in other datasets, preferably prospective cohorts of patients, is recommended. Third, the methods used to identify poststroke AF were not standardized in all patients and therefore biases in the measurement of the outcome might exist. Nevertheless, this study reflected real-world clinical practice where stroke patients are generally diagnosed with AF using 12-lead ECG or 24-h Holter ECG. Fourth, the CHASE-LESS score may have performed better than other scores in this cohort because it was developed in a population from Taiwan, with similar sociodemographic and ethnic characteristics.

## Conclusion

Of the eight risk scores validated in this cohort, the CHASE-LESS and AS5F scores demonstrated adequate discrimination and calibration for predicting AFDAS. These two simple risk scores may help refine the patient population for whom intensive AF monitoring is most likely to be beneficial. Further comparisons in AIS cohorts with different characteristics are needed.

## Data Availability Statement

The data analyzed in this study is subject to the following licenses/restrictions: The data used in this study cannot be made available because of restrictions regarding the use of EMR data. Requests to access these datasets should be directed to S-FS, richard.sfsung@gmail.com.

## Ethics Statement

The studies involving human participants were reviewed and approved by the Ditmanson Medical Foundation Chia-Yi Christian Hospital Institutional Review Board. Written informed consent for participation was not required for this study in accordance with the national legislation and the institutional requirements.

## Author Contributions

C-YH, H-MK, and S-FS contributed to the conception and design of the study. K-LS and S-FS organized the database and performed the statistical analysis. C-YH and H-MK wrote the first draft of the manuscript. K-LS, LS, S-FS, and S-JL wrote the sections of the manuscript. All authors contributed to manuscript revision, read, and approved the submitted version.

## Conflict of Interest

The authors declare that the research was conducted in the absence of any commercial or financial relationships that could be construed as a potential conflict of interest.

## Publisher’s Note

All claims expressed in this article are solely those of the authors and do not necessarily represent those of their affiliated organizations, or those of the publisher, the editors and the reviewers. Any product that may be evaluated in this article, or claim that may be made by its manufacturer, is not guaranteed or endorsed by the publisher.
