# Supplementary material for: Validation of Risk Scores for Predicting Atrial Fibrillation Detected After Stroke Based on an Electronic Medical Record Algorithm: A Registry-Claims-Electronic Medical Record Linked Data Study
Source: Front Cardiovasc Med. 2022 Apr 29;9:888240. doi: 10.3389/fcvm.2022.888240 (PMC9098928; doi:10.3389/fcvm.2022.888240)
Supplement: Supplementary file 1 [file Data_Sheet_1.pdf]

Supplementary Material

Supplementary Table 1. Derivation or validation of risk scores for predicting atrial fibrillation detected after stroke.

Risk score	Year	Components	AF ascertainment	C-index (95% CI)
Risk scores investigated in this study				
AS5F (1)	2019	Age, NIHSS	Holter monitoring (72 hours or more)	Derivation<br>0.78<br>Internal validation<br>0.78<br>External validation<br>1. 0.752<br>2. 0.765
AS5F (2)	2020	Age, NIHSS	ICD-9 codes	External validation<br>0.709 (0.680–0.738)
C_2HEST (3)	2019	Age, HTN, COPD, CAD, systolic HF, thyroid disease	ICD-10 codes	External validation<br>0.734 (0.732–0.736)
$CHADS_2$ (4)	2015	Age, CHF, HTN, DM, prior stroke	ICD-10 codes	External validation<br>0.700 (0.696–0.706)
$CHADS_2$ (5)	2017	Age, CHF, HTN, DM, prior stroke	6-day Holter monitoring	External validation<br>0.54 (0.46–0.61)
$CHADS_2$ (6)	2018	Age, CHF, HTN, DM, prior stroke	ICD-9 codes	External validation<br>1. 0.558 (0.538–0.577)<br>2. 0.597 (0.577–0.618)

$CHADS_2$ (1)	2019	Age, CHF, HTN, DM, prior stroke	Holter monitoring (72 hours or more)	External validation:<br>1. 0.607<br>2. 0.629
$CHADS_2$ (2)	2020	Age, CHF, HTN, DM, prior stroke	ICD-9 codes	External validation<br>0.536 (0.505–0.567)
$CHADS_2$ (7)	2020	Age, CHF, HTN, DM, prior stroke	ICD-9 codes	External validation<br>1. 0.600 (0.596–0.604)<br>2. 0.576 (0.568–0.583)
CHA_2DS_2-VASc (8)	2014	Age, sex, CHF, HTN, DM, prior stroke, vascular disease	ICD-10 codes	NA
CHA_2DS_2-VASc (4)	2015	Age, sex, CHF, HTN, DM, prior stroke, vascular disease	ICD-10 codes	External Validation<br>0.706 (0.702–0.710)
CHA_2DS_2-VASc (5)	2017	Age, sex, CHF, HTN, DM, prior stroke, vascular disease	6-day Holter monitoring	External Validation<br>0.58 (0.50–0.65)
CHA_2DS_2-VASc (6)	2018	Age, sex, CHF, HTN, DM, prior stroke, vascular disease	ICD-9 codes	External Validation<br>1. 0.603 (0.584–0.622)<br>2. 0.644 (0.624–0.665)
CHA_2DS_2-VASc (3)	2019	Age, sex, CHF, HTN, DM, prior stroke, vascular disease	ICD-10 codes	External Validation<br>0.703 (0.701–0.704)
CHA_2DS_2-VASc (2)	2020	Age, sex, CHF, HTN, DM, prior stroke, vascular disease	ICD-9 codes	External validation<br>0.578 (0.547–0.609)

CHA_2DS_2-VASc (7)	2020	Age, sex, CHF, HTN, DM, prior stroke, vascular disease	ICD-9 codes	External validation<br>1. 0.636 (0.632–0.640)<br>2. 0.607 (0.600–0.615)
CHASE-LESS (2)	2020	Age, NIHSS, CAD, CHF, hyperlipidemia, DM, prior stroke or TIA	ICD-9 codes	Derivation<br>0.730 (0.711–0.748)<br>Internal validation<br>0.732 (0.703–0.761)
HATCH (6)	2018	Age, HTN, COPD, prior stroke or TIA, CHF	ICD-9 codes	External validation<br>1. 0.612 (0.692–0.730)<br>2. 0.653 (0.633–0.674)
HAVOC (9)	2017	Age, HTN, valvular disease, PAD, obesity, CHF, CAD	ICD-9 codes	Derivation<br>0.77<br>Internal validation<br>0.77
Re-CHARGE-AF (10)	2021	Age, race, height, weight, SBP, DBP, smoking, antihypertensive medication use, DM, CHF, MI	ICD-9 or ICD-10 codes	Derivation<br>0.74 (0.68–0.79)<br>Internal validation<br>0.70 (0.65–0.75)
Risk scores not investigated in this study				
ACT (11)	2018	Age, cardiac disease, troponin	Holter monitoring	Derivation<br>0.795

ACTEL (12)	2020	Age, LAE, hypercholesterolemia, ≥ mild to moderate tricuspid regurgitation, left ventricular end diastolic volume	Continuous ECG monitoring for 5 days or more	Derivation 0.80 (0.73–0.87)
AF-ESUS (14)	2021	Age, LAE, HTN, non-stenotic carotid plaque, LVH, reduced LVEF <35%, supraventricular extrasystole, subcortical infarct	Clinical AF, or ECG during follow-up visits	Derivation 0.848 (0.799–0.869)
AF-ESUS (15)	2021	Age, LAE, HTN, non-stenotic carotid plaque, LVH, reduced LVEF <35%, supraventricular extrasystole, subcortical infarct	Implantable loop recorder	NA
ASAS (13)	2014	Age, LAE, NIHSS	Previous history, ECG, Holter monitoring, or cardiac telemetry during hospitalization	Derivation 0.79 (0.71–0.86) Internal validation 0.76 (0.69–0.83)
Brown ESUS-AF (16)	2018	Age, LAE	30-day ECG monitoring, implantable cardiac monitor	Derivation 0.726
CHARGE-AF (17)	2021	Age, race, height, weight, SBP, DBP, smoking, antihypertensive medication, DM, CHF, myocardial infarction	1. 24 to 48-hour Holter/ECG monitor, 2. 2 to 4-week event/patch monitor, or 3. implantable loop recorder	NA
CHARGE-AF (10)	2021	Age, race, height, weight, SBP, DBP, smoking, antihypertensive medication use, DM, CHF, MI	ICD-9 or ICD-10 codes	External validation 0.64 (0.57–0.70)

Chen et al (7)	2020	1. Non-diabetics: Age, HTN, CAD, CHF, COPD, female sex, gout, statin use<br>2. Diabetics: Age, HTN, CAD, CHF, COPD, CKD, statin use	ICD-9 codes	Derivation<br>1. 0.671 (0.667–0.675)<br>2. 0.630 (0.623–0.638)
Framingham risk score (18)	2019	Age, BMI, SBP, antihypertensive medication use, PR interval, age at which significant cardiac murmur developed, age of HF	ICD-10 codes	External validation<br>0.720 (0.718–0.722)
iPAB (19)	2015	BNP, prior arrhythmia or use of antiarrhythmic agents	Bedside continuous ECG monitoring, 24-hour Holter monitoring	Derivation<br>0.90 (0.85–0.94)<br>External validation<br>0.94 (0.89–0.98)
LADS scoring system (20)	2011	Age, LAE, stroke versus TIA, smoking	Telemetry	NA
Liu et al (5)	2017	1. CHA_2DS_2-VASc plus neuroimaging findings<br>2. CHADS2 Scores plus neuroimaging findings	6-day Holter monitoring	Derivation<br>1. 0.74 (0.67–0.81)<br>2. 0.75 (0.68–0.82)
MrWALLETS (21)	2017	Age, LAE, mild-to-moderate mitral regurgitation, white matter disease, stroke size on neuroimaging, left ventricular end-diastolic volume, ≥ moderate tricuspid regurgitation, carotid stenosis ≥50%	Continuous ECG monitoring	Derivation<br>0.89 (0.83–0.95)
Seo et al (22)	2016	Age, LAE, free fatty acid level, triglyceride level, susceptibility vessel sign, hemorrhagic transformation, cortical involvement	3-day continuous ECG monitoring, 24-hour Holter monitoring in 43.4% of patients	Derivation<br>0.908 (0.887–0.930)

STAF (23)	2009	Age, LAE, NIHSS, absence of vascular etiology	Previous history, initial ECG, ECG monitoring during hospitalization, and 24-hour Holter ECG	Derivation 0.94 (0.92–0.96)
STAF (19)	2015	Age, LAE, NIHSS, absence of vascular etiology	Bedside continuous ECG monitoring, 24-hour Holter monitoring	External Validation 0.77 (0.66–0.88)

AF, atrial fibrillation; BMI, body mass index; BNP, brain natriuretic peptide; CAD, coronary artery disease; CHF, congestive heart failure; CI, confidence interval; CKD, chronic kidney disease; COPD, chronic obstructive pulmonary disease; DBP, diastolic blood pressure; DM, diabetes mellitus, ECG, electrocardiography; HF, heart failure; HTN, hypertension; ICD, International Classification of Diseases; LAE, left atrial enlargement; LVEF, left ventricular ejection fraction; LVH, left ventricular hypertrophy; MI, myocardial infarction; NA, not available; NIHSS, National Institutes of Health Stroke Scale; SBP, systolic blood pressure; TIA, transient ischemic attack;

Supplementary Table 2. Definition of various algorithms to identify AF status.

Algorithm	Definition
Component	
AF-1	Any positive mention of "atrial fibrillation", "AF", "Afib", or "PAF" in ECG reports
AF-2	Any positive mention of "atrial fibrillation", "AF", "Afib", or "PAF" in echocardiography reports
AF-3	Any positive mention of "atrial fibrillation", "AF", "Afib", or "PAF" in physicians' notes
AF-4	Diagnostic codes for AF (ICD-9-CM 427.31 or ICD-10-CM I48.91)
AF-5	On antiarrhythmic medications (amiodarone, dronedarone, or propafenone)
AF-6	On oral anticoagulants (warfarin, apixaban, edoxaban, rivaroxaban, or dabigatran)
Composite	
AF-A	AF-1 or AF-4
AF-B	AF-1 or AF-3
AF-C	AF-3 or AF-4
AF-D	AF-1 or AF-2 or AF-4
AF-E	AF-1 or AF-3 or AF-4
AF-F	AF-1 or AF-2 or AF-3 or AF-4
AF-G	AF-1 or AF-2 or AF-3 or AF-5
AF-H	AF-1 or AF-2 or AF-3 or AF-6
AF-I	AF-1 or AF-2 or AF-3 or AF-5 or AF-6
AF-J	AF-1 or AF-2 or AF-3 or AF-4 or AF-5 or AF-6

AF, atrial fibrillation; ECG, electrocardiography; ICD-9-CM, International Classification of Diseases, Ninth Revision, Clinical Modification; ICD-10-CM, International Classification of Diseases, Tenth Revision, Clinical Modification.

Supplementary Table 3. Risk scores for predicting AFDAS.

Variable	AS5F	C_2HEST	$CHADS_2$	CHA_2DS_2-VASc	CHASE-LESS	HATCH	HAVOC	Re-CHARGE-AF
Age	+0.76 per year				+1 per decade			+0.286 per 5 years
65–74				+1				
≥75		+2	+1	+2		+1	+2	
Female				+1				
Hypertension		+1	+1	+1		+1	+2	
SBP								+0.023 per 20 mmHg
DBP								-0.116 per 10 mmHg
Antihypertensive medication use								+0.004
Diabetes mellitus			+1	+1	-1			-0.488
Hyperlipidemia					-1			
CAD		+1			+1		+2	+0.282
PAD							+1	
CAD or PAD				+1				
CHF		+2	+1	+1	+1	+2	+4	+0.627
Prior stroke or TIA			+2	+2	-1	+2		

Valve disease					+2	
COPD		+1		+1		
Hyperthyroidism		+1				
Smoking (current)						
Obesity (BMI >30)					+1	
Height						-0.133 per 10 cm
Weight						0.421 per 15 kg
NIHSS						
≤5	+9					
6–13	+21		+1			
≥14	+21		+4			

AF, atrial fibrillation; AFDAS, atrial fibrillation detected after stroke; BMI, body mass index; CAD, coronary artery disease; CHF, congestive heart failure; COPD, chronic obstructive pulmonary disease; DBP, diastolic blood pressure; NIHSS, National Institutes of Health Stroke Scale; PAD, peripheral artery disease; SBP, systolic blood pressure; TIA, transient ischemic attack.

Supplementary Table 4. Performance of various algorithms to identify AF status.

Algorithm	Sen	Spe	PPV	NPV	AUC (95% CI)
Component					
AF-1	89.3%	100.0%	100.0%	97.9%	0.947 (0.923–0.970)
AF-2	50.3%	100.0%	100.0%	90.8%	0.751 (0.714–0.789)
AF-3	96.4%	97.6%	89.1%	99.3%	0.970 (0.955–0.985)
AF-4	78.7%	99.8%	98.5%	95.8%	0.892 (0.861–0.923)
AF-5	33.1%	99.2%	88.9%	87.9%	0.661 (0.626–0.697)
AF-6	42.6%	98.9%	88.9%	89.4%	0.708 (0.670–0.745)
Composite					
AF-A	95.9%	99.8%	98.8%	99.2%	0.978 (0.963–0.993)
AF-B	100.0%	97.6%	89.4%	100.0%	0.988 (0.983–0.993)
AF-C	97.0%	97.5%	88.6%	99.4%	0.973 (0.959–0.986)
AF-D	98.2%	99.8%	98.8%	99.6%	0.990 (0.980–1.000)
AF-E	100.0%	97.5%	88.9%	100.0%	0.987 (0.982–0.993)
AF-F	100.0%	97.5%	88.9%	100.0%	0.987 (0.982–0.993)
AF-G	100.0%	96.8%	86.2%	100.0%	0.984 (0.978–0.990)
AF-H	100.0%	96.5%	85.4%	100.0%	0.983 (0.976–0.989)
AF-I	100.0%	95.7%	82.4%	100.0%	0.978 (0.971–0.985)
AF-J	100.0%	95.7%	82.4%	100.0%	0.978 (0.971–0.985)

AF, atrial fibrillation; AUC, area under the receiver operating characteristic curve; CI, confidence interval; NPV, negative predictive value; PPV, positive predictive value; Sen, sensitivity, Spe, specificity.

Supplementary Table 5. ***P*** **values of pairwise comparisons of C-indices between risk scores.**

Risk score	C_2HEST	$CHADS_2$	CHA_2DS_2-VASc	CHASE-LESS	HATCH	HAVOC	Re-CHARGE-AF
AS5F	<0.001	<0.001	<0.001	0.223	<0.001	<0.001	0.002
C_2HEST	-	<0.001	0.252	<0.001	<0.001	0.009	0.008
$CHADS_2$	-	-	<0.001	<0.001	0.002	<0.001	<0.001
CHA_2DS_2-VASc	-	-	-	<0.001	0.001	0.683	0.002
CHASE-LESS	-	-	-	-	<0.001	<0.001	<0.001
HATCH	-	-	-	-	-	0.027	<0.001
HAVOC	-	-	-	-	-	-	<0.001

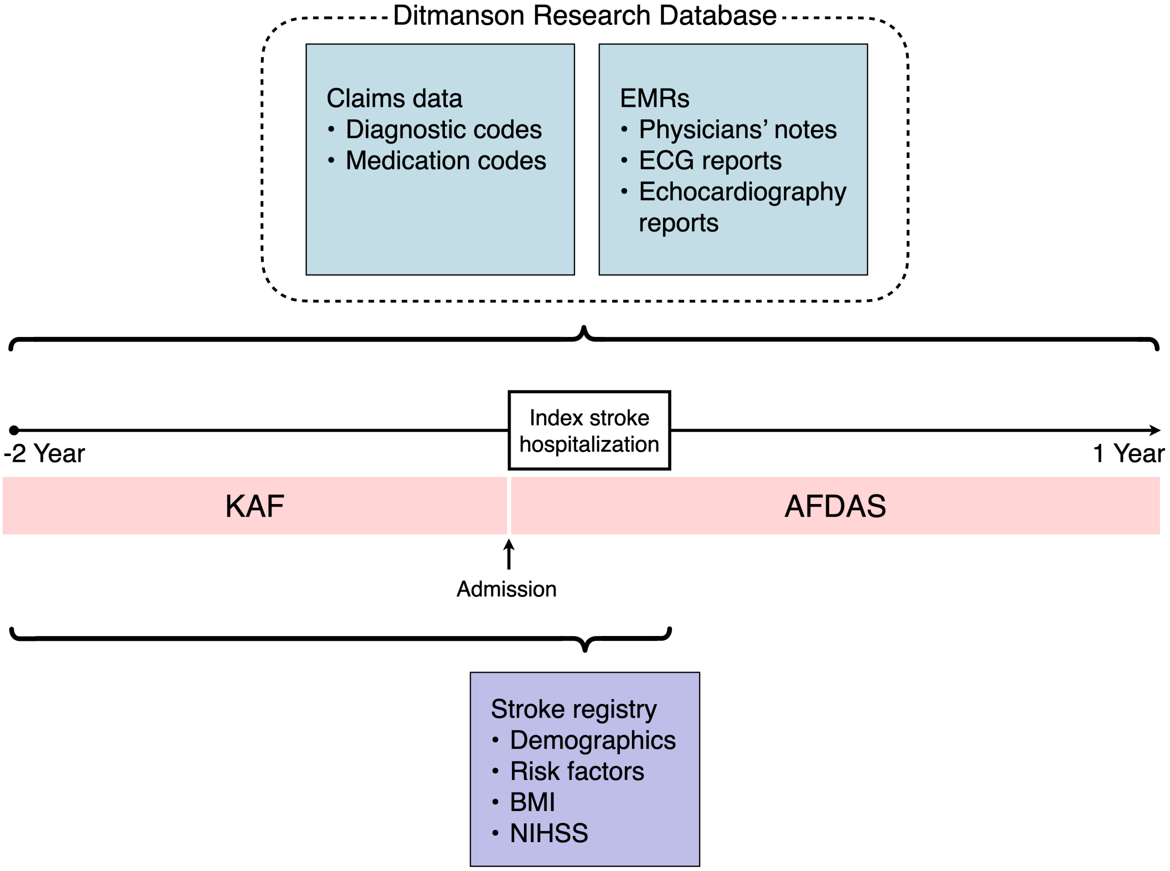

Supplementary Figure 1. Schematic illustration of the study design and data sources. AFDAS, atrial fibrillation detected after stroke; BMI, body mass index; ECG, electrocardiography; EMRs, electronic medical records; KAF, known atrial fibrillation; NIHSS, National Institutes of Health Stroke Scale.

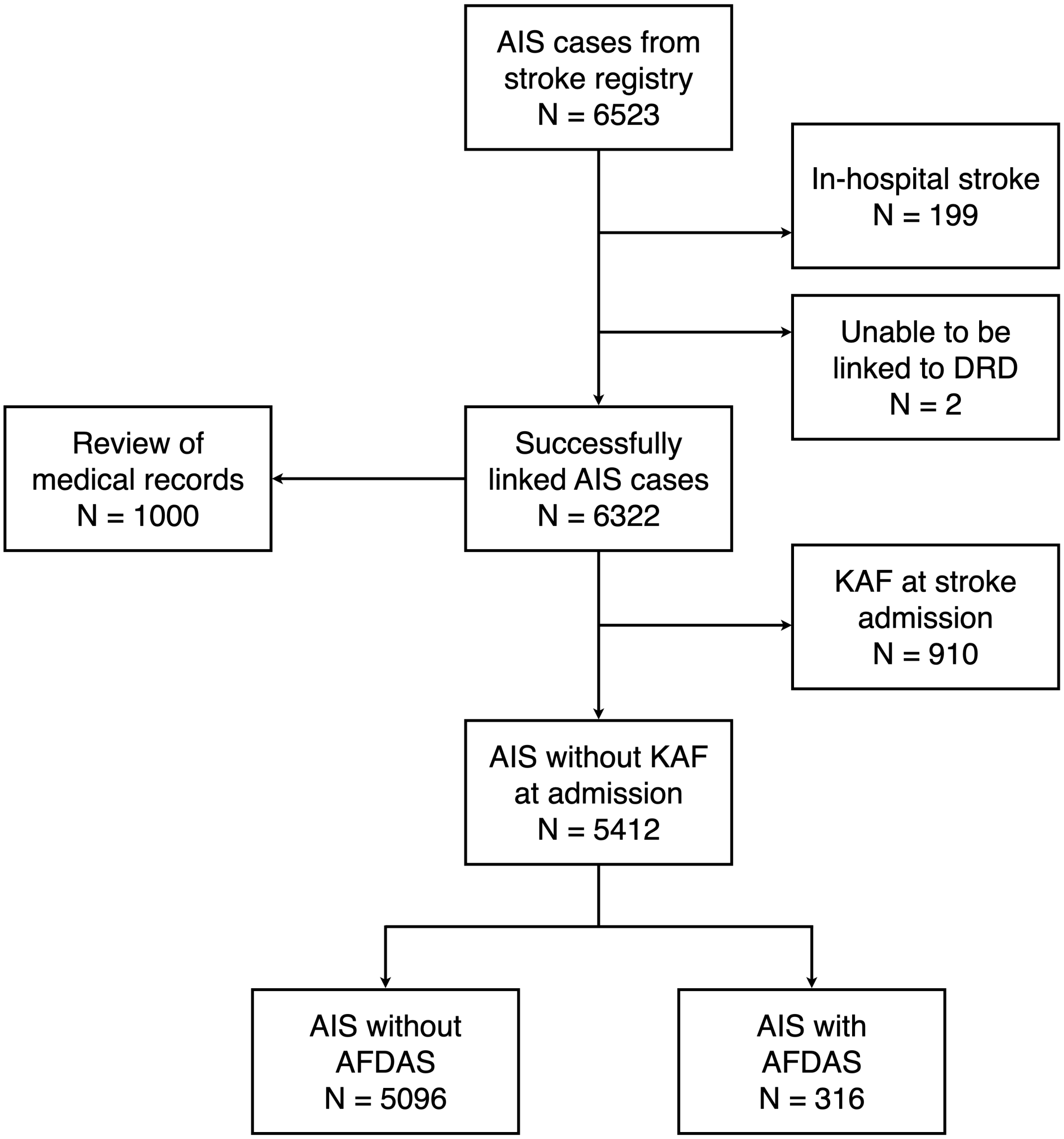

Supplementary Figure 2. Flowchart of study population selection. AFDAS, atrial fibrillation detected after stroke; AIS, acute ischemic stroke; DRD, Ditmanson Research Database; KAF, known atrial fibrillation.

Supplementary References

1. Uphaus T, Weber-Krüger M, Grond M, Toenges G, Jahn-Eimermacher A, Jauss M, et al. Development and validation of a score to detect paroxysmal atrial fibrillation after stroke. *Neurology.* (2019) 92:e115–e124. doi: 10.1212/wnl.0000000000006727

2. Hsieh C-Y, Lee C-H, Sung S-F. Development of a novel score to predict newly diagnosed atrial fibrillation after ischemic stroke: The CHASE-LESS score. *Atherosclerosis.* (2020) 295:1–7. doi: 10.1016/j.atherosclerosis.2020.01.003

3. Li Y, Bisson A, Bodin A, Herbert J, Grammatico-Guillon L, Joung B, et al. C_2HEST Score and Prediction of Incident Atrial Fibrillation in Poststroke Patients: A French Nationwide Study. *J Am Heart Assoc.* (2019) 8:e012546. doi: 10.1161/jaha.119.012546

4. Fauchier L, Clementy N, Pelade C, Collignon C, Nicolle E, Lip GYH. Patients With Ischemic Stroke and Incident Atrial Fibrillation: A Nationwide Cohort Study. *Stroke.* (2015) 46:2432–2437. doi: 10.1161/strokeaha.115.010270

5. Liu R, Yang X, Li S, Jiang Y, Wang Y, Wang Y. Novel composite scoring system to predict unknown atrial fibrillation in acute ischemic stroke patients. *Brain Res.* (2017) 1674:36–41. doi: 10.1016/j.brainres.2017.08.005

6. Hsieh C-Y, Lee C-H, Wu DP, Sung S-F. Prediction of new-onset atrial fibrillation after first-ever ischemic stroke: A comparison of CHADS2, CHA2DS2-VASc and HATCH scores and the added value of stroke severity. *Atherosclerosis.* (2018) 272:73–79. doi: 10.1016/j.atherosclerosis.2018.03.024

7. Chen Y-L, Wang H-T, Chen H-C, Liu W-H, Hsueh S, Chung W-J, et al. A risk stratification scoring system for new-onset atrial fibrillation after ischemic stroke. *Medicine.* (2020) 99:e20881. doi: 10.1097/md.0000000000020881

8. Friberg L, Rosenqvist M, Lindgren A, Terént A, Norrving B, Asplund K. High prevalence of atrial fibrillation among patients with ischemic stroke. *Stroke.* (2014) 45:2599–2605. doi: 10.1161/strokeaha.114.006070

9. Kwong C, Ling AY, Crawford MH, Zhao SX, Shah NH. A Clinical Score for Predicting Atrial Fibrillation in Patients with Cryptogenic Stroke or Transient Ischemic Attack. *Cardiology.* (2017) 138:133–140. doi: 10.1159/000476030

10. Ashburner JM, Wang X, Li X, Khurshid S, Ko D, Lipsanopoulos AT, et al. Re-CHARGE-AF: Recalibration of the CHARGE-AF Model for Atrial Fibrillation Risk Prediction in Patients With Acute Stroke. *J Am Heart Assoc.* (2021) 10:e022363. doi: 10.1161/jaha.121.022363

11. Naess H, Andreassen UW. A score for paroxysmal atrial fibrillation in acute ischemic stroke. *Int J Stroke.* (2018) 13:496–502. doi: 10.1177/1747493017724623

12. Muscari A, Barone P, Faccioli L, Ghinelli M, Trossello MP, Puddu GM, et al. Usefulness of the ACTEL Score to Predict Atrial Fibrillation in Patients with Cryptogenic Stroke. *Cardiology.* (2020) 145:168–177. doi: 10.1159/000505262

13. Figueiredo MM, Rodrigues ACT, Alves MB, Neto MC, Silva GS. Score for atrial fibrillation detection in acute stroke and transient ischemic attack patients in a Brazilian population: the acute stroke atrial fibrillation scoring system. *Clinics (Sao Paulo).* (2014) 69:241–246. doi: 10.6061/clinics/2014(04)04

14. Ntaios G, Perlepe K, Lambrou D, Sirimarco G, Strambo D, Eskandari A, et al. Identification of patients with embolic stroke of undetermined source and low risk of new incident atrial fibrillation: The AF-ESUS score. *Int J Stroke.* (2020) 16:29–38. doi: 10.1177/1747493020925281

15. Kitsiou A, Sagris D, Schäbitz W-R, Ntaios G. Validation of the AF-ESUS score to identify patients with embolic stroke of undetermined source and low risk of device-detected atrial fibrillation. *Eur J Intern Med.* (2021) 89:135–136. doi: 10.1016/j.ejim.2021.04.003

16. Ricci B, Chang AD, Hemendinger M, Dakay K, Cutting S, Burton T, et al. A Simple Score That Predicts Paroxysmal Atrial Fibrillation on Outpatient Cardiac Monitoring after Embolic Stroke of Unknown Source. *J Stroke Cerebrovasc Dis.* (2018) 27:1692–1696. doi: 10.1016/j.jstrokecerebrovasdis.2018.01.028

17. Khurshid S, Li X, Ashburner JM, Lipsanopoulos ATT, Lee PR, Lin AK, et al. Usefulness of Rhythm Monitoring Following Acute Ischemic Stroke. *Am J Cardiol.* (2021) doi: 10.1016/j.amjcard.2021.01.038

18. Schnabel RB, Sullivan LM, Levy D, Pencina MJ, Massaro JM, D’Agostino RB, et al. Development of a risk score for atrial fibrillation (Framingham Heart Study): a community-based cohort study. *Lancet.* (2009) 373:739–745. doi: 10.1016/s0140-6736(09)60443-8

19. Yoshioka K, Watanabe K, Zeniya S, Ito Y, Hizume M, Kanazawa T, et al. A Score for Predicting Paroxysmal Atrial Fibrillation in Acute Stroke Patients: iPAB Score. *J Stroke Cerebrovasc Dis.* (2015) 24:2263–2269. doi: 10.1016/j.jstrokecerebrovasdis.2015.06.019

20. Malik S, Hicks WJ, Schultz L, Penstone P, Gardner J, Katramados AM, et al. Development of a scoring system for atrial fibrillation in acute stroke and transient ischemic attack patients: The LADS scoring system. *J Neurol Sci.* (2011) 301:27–30. doi: 10.1016/j.jns.2010.11.011

21. Muscari A, Bonfiglioli A, Faccioli L, Ghinelli M, Magalotti D, Manzetto F, et al. Usefulness of the MrWALLETS Scoring System to Predict First Diagnosed Atrial Fibrillation in Patients With Ischemic Stroke. *Am J Cardiol.* (2017) 119:1023–1029. doi: 10.1016/j.amjcard.2016.12.009

22. Seo W-K, Kang S-H, Jung J-M, Choi J-Y, Oh K. Novel composite score to predict atrial Fibrillation in acute stroke patients: AF predicting score in acute stroke. *Int J Cardiol.* (2016) 209:184–189. doi: 10.1016/j.ijcard.2016.02.002

23. Suissa L, Bertora D, Lachaud S, Mahagne MH. Score for the targeting of atrial fibrillation (STAF): a new approach to the detection of atrial fibrillation in the secondary prevention of ischemic stroke. *Stroke.* (2009) 40:2866–2868. doi: 10.1161/strokeaha.109.552679
